# Carotid Body Tumor in a 26-Year-Old Male Patient Managed With Preoperative Embolization

**DOI:** 10.7759/cureus.49917

**Published:** 2023-12-04

**Authors:** Nikolaos Avgerinos, Ilias Avgerinos, Theodore Troupis, Dimosthenis Chrysikos, Sotirios Georgopoulos

**Affiliations:** 1 Anatomy, National and Kapodistrian University of Athens School of Medicine, Athens, GRC; 2 Vascular Surgery, Laiko General Hospital of Athens, Athens, GRC; 3 Surgery, National and Kapodistrian University of Athens School of Medicine, Athens, GRC

**Keywords:** preoperative embolization, carotid body tumor preoperative embolization, carotid body tumors, carotid body tumor diagnosis, carotid body tumor pre-operative embolization, hemodectoma, carotid body paragaglioma, carotid body tumor

## Abstract

Carotid body tumors are rare, highly vascularized neuroendocrine tumors that arise near the bifurcation of the common carotid artery (CCA). Controversy exists in the management of those tumors about whether preoperative embolization facilitates surgical excision and decreases perioperative complication risk. We present the case of a 26-year-old patient with a carotid body tumor manifesting as a painless pulsatile mass in the anterior triangle over the left side of the neck and provide details of the preoperative diagnostic steps. Treatment included preoperative embolization of the tumor followed by surgical excision after 48 hours to safely address this rare pathology, resulting in a favorable outcome for the patient.

## Introduction

Carotid body tumors are rare, highly vascularized neuroendocrine neoplasms located near the common carotid artery (CCA) bifurcation in the carotid bulb, constituting just 0.6% of all head and neck tumors, with an incidence rate of one per 30,000 for Caucasians and 1.6/10,000 annually [1, 2]. Tumors arising from the cells of the carotid bulb that function as chemoreceptors [3] are also called carotid body paragangliomas or chemodectomas [1,4-6]. Carotid body tumors account for 65% of all head and neck paragangliomas and are the most common endocrine neoplasms in the cervical region [4,7]. Usually, they appear between the fifth and seventh decade of life, with an increased prevalence in females, with a ratio of 1.9:1 compared to men [6]. Three etiological types have been described in the literature [4], with the familial subtype presenting an autosomal dominant pattern accounting for 10% of carotid body tumors [4].

Carotid body tumors are usually benign; however, the rate of malignant transformation has been reported to be 6% [2]. Malignancy is commonly indicated by the presence of metastasis to non-endocrine tissues [2]. Therefore, a full-body computed tomography angiography (CTA) should be used to investigate this possibility both before surgery and during follow-up.

Surgical resection constitutes the definitive management of carotid body tumors. Tumor size has been directly associated with operative complications such as cranial nerve injury [7]. Thus, early surgical excision upon the establishment of a diagnosis has been strongly recommended [1,4,7-12]. 

In 1971, Shamblin devised an algorithm that sorted carotid body tumors in the context of carotid artery involvement [6]. Shamblin type I tumors are small, do not splay the carotid bifurcation and are generally resected without difficulty. Shamblin type II tumors are larger, considerably splay the carotid bifurcation, and adhere to the carotid arteries, but only partially surround them. Shamblin type III tumors are large and encase the carotid arteries, with cranial nerves commonly adhered to or incorporated into the tumor [6, 9].

To benefit the surgical procedure, preoperative selective embolization performed 24-48 hours prior to surgery has commonly been implemented as a preoperative treatment strategy aimed at decreasing tumor size, minimizing intraoperative blood loss, and reducing cranial nerve injury incidence [13].

We present the case of a 26-year-old male patient with an asymptomatic, painless palpable mass in the left side of the neck; management included preoperative embolization of the tumor, which preceded 48 hours of definitive surgical excision.

## Case presentation

A 26-year-old male patient of Arabian descent presented at our clinic for a scheduled appointment due to a palpable mass in the left side of the neck, which gradually increased in size over the last six months, without complaining of any other associated symptomatology. The patient’s medical history was clear, and he was a non-smoker with no known allergies or family history of relevant pathologies.

**Figure 1:**
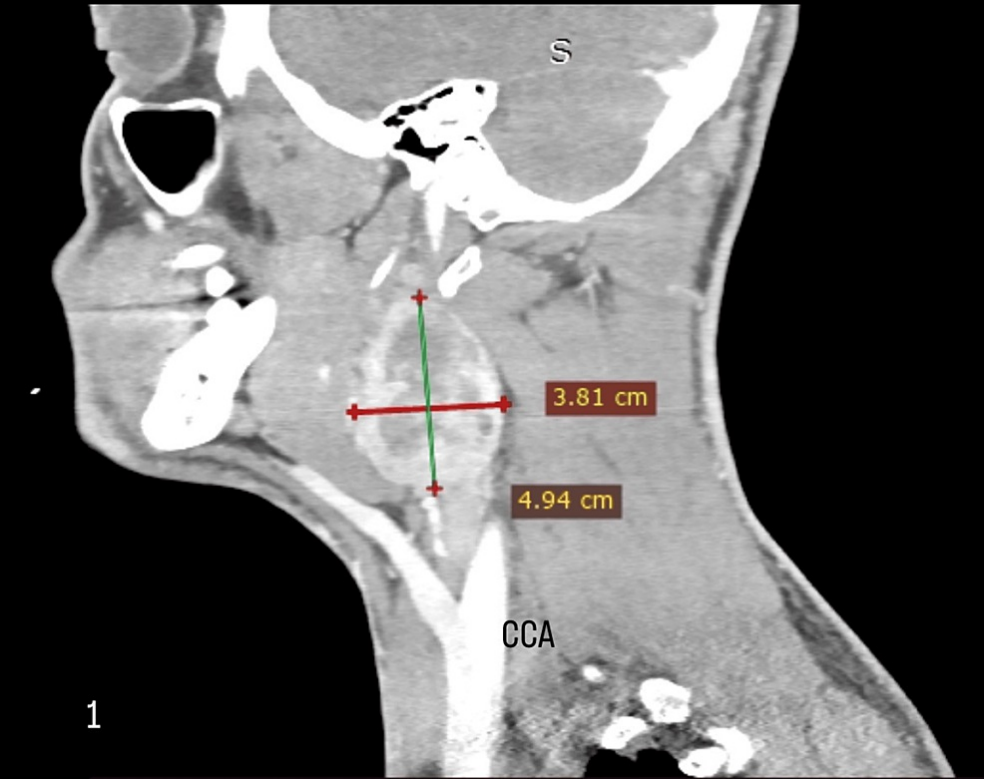
Computed tomography angiography of the neck shows an oval-shaped, well-defined mass located in the left carotid bifurcation, measuring 3.8 cm x 4.94 cm, highly enhanced by the contrast agent. CCA: common carotid artery

Clinical examination revealed a pulsatile, firm, painless mass in the anterior triangle of the left side of the neck, characteristically mobile in the horizontal plane but not in the vertical plane (positive Fontaine’s sign). The next step in the diagnostic approach included a CTA of the neck. An oval-shaped, well-defined mass located in the left carotid bifurcation, measuring 3.8 cm x 4.94 cm, was highly enhanced by the contrast agent (Figure 1).

**Figure 2:**
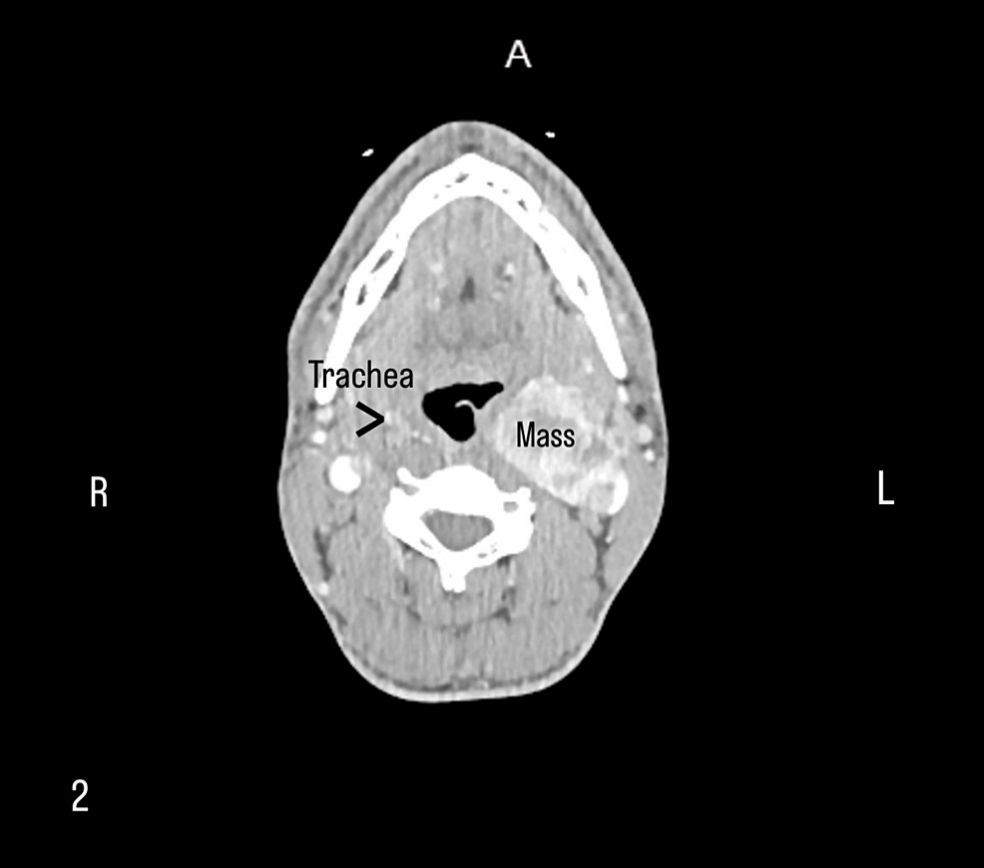
Computed tomography angiography of the neck shows partial compression of the trachea by the tumor.

The mass was shown to displace the left internal carotid artery posteriorly and the left external carotid artery (ECA) anteriorly, creating the characteristic lyre sign. The CTA also revealed partial compression of the trachea by the tumor (Figure 2).

The CTA findings sorted the tumor as type II according to the Shamblin classification [6]. Laboratory workup concerning the endocrinological activity of the tumor was within normal ranges.

Our therapeutic decision was the surgical resection of the mass. However, the increased tumor volume and the fact that it was shown to present rich vascularization led us to perform preoperative embolization.

**Figure 3:**
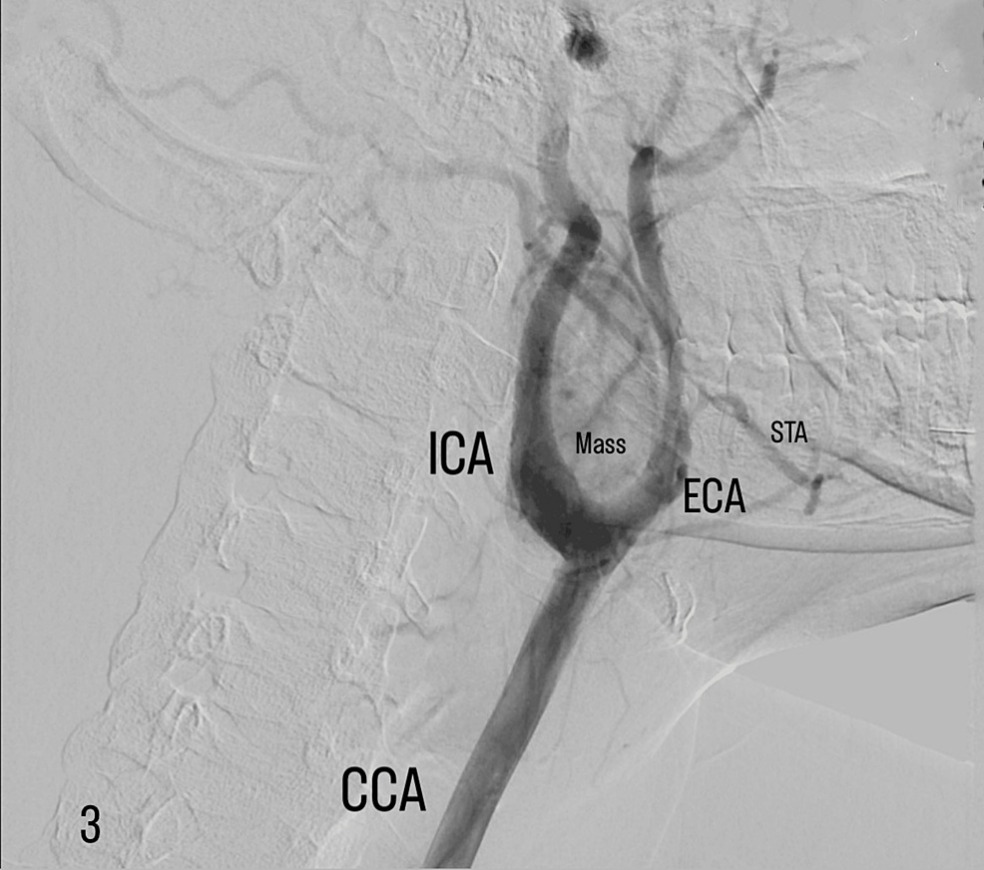
Digital subtraction angiography for preoperative tumor embolization shows the characteristic splaying of the two main left carotid branches (lyre sign). Note the rich blood supply to the mass, with feeding vessels originating from the left external carotid artery. CCA: common carotid artery; ECA: external carotid artery; ICA: internal carotid artery

Preoperative embolization was carried out under local anesthesia by interventional radiologists at the angiographic suite. Intraarterial access was obtained through the right common femoral artery. A 7-Fr x 90-cm-long introducing sheath was inserted in the thoracic descending aorta. A digital subtraction angiogram (DSA) was performed, which again confirmed the characteristic splaying of the two main left carotid branches (lyre sign) and showed rich blood supply to the mass with feeding vessels originating from the left ECA (Figure 3).

**Figure 4:**
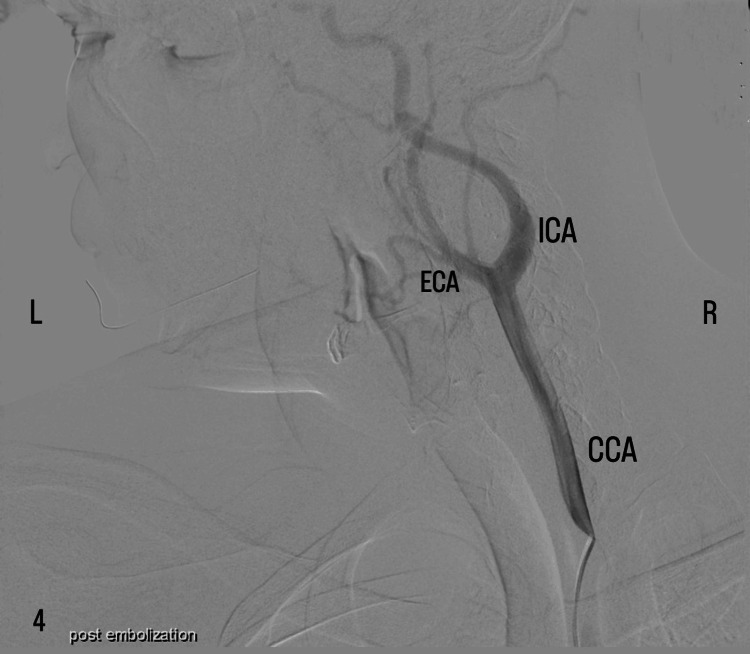
Post-embolization angiographic assessment (DSA) shows the complete elimination of uptake of the contrast agent in the mass, implying adequate embolization. Note the passage of contrast agent at the carotid bifurcation arteries, signifying their patency at the end of the procedure. CCA: common carotid artery; ECA: external carotid artery; ICA: internal carotid artery; DSA: digital subtraction angiography

Selective catheterization of the left ECA was performed, followed by super-selective catheterization of the tumor-feeding vessels. Embolization included the infusion of a tris-acryl gelatin microsphere (TAGM) agent using a microcatheter. The final angiographic assessment showed a complete and successful elimination of the uptake of contrast agents in the mass. The patency of the carotid bifurcation was confirmed by the passage of a contrast agent at the two main left carotid branches at the end of the procedure (Figure 4).

The patient’s post-embolization course was uneventful, and he remained hospitalized until surgical resection of the tumor.

Forty-eight hours later, the surgical resection of the tumor was carried out. The procedure commenced with the patient under general anesthesia, and the head was turned to the right side to facilitate exposure of the left neck region.

The left lower calf was also cleaned and prepped in case an interposition venous graft from the saphenous was necessary to repair any damage to the carotid arteries. A single dose of second-generation cephalosporin was administered intravenously 30 minutes before anesthesia induction.

The vessels of the left carotid bifurcation were exposed using a standard anterior approach through a longitudinal incision over the anterior border of the sternocleidomastoid muscle. A 4.1 cm tumor, approximately 20% smaller in size compared to the dimensions measured on CTA prior to embolization, was resected and sent for histopathological examination. Less than 100 ml of blood was lost during the entire operation. No injuries to the carotid bifurcation occurred during the operation, and no cranial nerve injury was noticed intraoperatively or after the patient awoke from anesthesia.

A 16-Fr drain was placed, and closure was performed by anatomical layers. Suturing included the use of a continuous pattern with absorbable sutures for the platysma (Ethicon-coated polyglactin 910, size 2-0 suture (Johnson & Johnson, New Brunswick, NJ)). Skin closure was performed using staples.

The postoperative course was uneventful; the drain was removed the next morning, and the patient was discharged from the hospital on the third postoperative day without any medication prescribed.

Histopathological examination confirmed the initial diagnosis of carotid body paraganglioma, and the mass was positive by immunohistochemistry for chromatografin, synaptophysin, S-GATA, and S-100.

The patient was instructed to visit our clinic at one-month, six-month, and 12-month intervals, and follow-up included clinical examination and a full-body CTA scan for the evaluation of tumor recurrence and metastasis. All the results were negative.

## Discussion

Medical history, clinical examination, and radiological imaging are fundamental for the diagnosis of carotid body tumors.

Studies have found that carotid body tumors clinically present as a painless, completely asymptomatic mass in the neck region [1,7,11,12]. On clinical examination and palpation, the mass is usually rubbery, firm, and incompressible, with a palpable thrill or bruit present, unless the tumor is very small in size [4,6]. The characteristic Fontaine's sign is positive when the tumor is laterally mobile but not vertically [4]. Less frequently presenting symptoms include dysphagia, hoarseness, and possibly tinnitus, indicating tumor pressure on the adjacent cranial nerve structures [1,4,7,13]. Transient ischemic attack or stroke, as well as neuroendocrine symptoms including headaches, palpitations, tachycardia, or flushing, have been reported in the literature, but with a very low incidence rate [1,4,6,11,12].

Doppler ultrasonography, CT scan, CTA, MRI, and magnetic resonance angiography (MRA) are the most widely employed modes for the diagnosis of carotid body tumors [4,7]. A soft tissue tumor seen at the level of the carotid bifurcation on either CT or MRI, splaying the internal and external carotid arteries and creating the characteristic lyre sign, constitutes the pathognomonic radiologic diagnostic sign [13]. A CTA or MRA and the high-resolution images they offer assist the surgeon in formulating the surgical strategy because of the excellent illustration of the vascular anatomy they provide [4,7]. Although MRI does not use ionizing radiation and often offers higher accuracy [6], the ability of CTA to measure tumor size and its association with bony structures seems to be a major advantage in surgical planning and perioperative complication risk prediction [10,11,14,15]. Aspiration needle biopsy has been reported in the literature for the diagnosis of carotid body tumors; however, we do not perform the procedure in our clinic because of the significantly increased risk of related complications, including massive hemorrhage, pseudoaneurysm formation, and carotid thrombosis [6].

An intraarterial DSA is routinely performed before surgery for preoperative planning, tumor feeding-vessel localization, and embolization [14]. Feeding vessels from the ECA, namely the ascending pharyngeal artery and its branches, have been described as the main vessels supplying carotid paragangliomas [11,12,14] with vessels from the internal carotid artery, vertebral artery, and superior thyroid artery also contributing to the intricate hypervascularization of the tumor [6].

A three-type system devised by Shamblin in 1971 is currently widely employed to define carotid body tumors and their involvement with the carotid arteries, which assists surgeons in formulating an operative plan and predicting significant perioperative complications [7,11,12,16] including blood loss and cranial nerve injury. A higher Shamblin grade is associated with an increased risk of such a possibility [10,11].

Surgical resection remains the definitive treatment of carotid body tumors concerning long-term recurrence and the possible prevention of malignancy [4,6]. Tumor size is considered the most significant factor associated with the incidence of perioperative cranial nerve injury [7], and early surgical excision upon diagnosis is recommended [1,4,7,9,11].

Radiation therapy as an alternative treatment or adjunct to surgery to decrease tumor size [4,7,11,13] and a conservative approach involving observation of the mass with regular MRI assessment have been described as alternatives; however, high rates of new or progressive cranial nerve (CN) deficits occur with the latter [4,11,13].

The increased blood network that these tumors present, along with their degree of carotid artery and cranial nerve involvement, makes the operation challenging for the surgeon [1]. Major postoperative complications associated with the excision of carotid body tumors include transient ischemic attack, CN damage, wound hematoma, and Horner syndrome. Studies have included cranial nerve XII (CNXII) or the hypoglossal nerve, cranial nerve X (CNX) or the vagus nerve, the recurrent laryngeal nerve, and the marginal mandibular branch of the facial nerve as the most commonly injured cranial nerves following surgery [8].

Attempting to make surgical excision easier for the surgeon by decreasing tumor size, decreasing tumor vascularization, and minimizing intraoperative blood loss, preoperative embolization of the tumor-feeding vessels has been proposed for the management of carotid body tumors [1,15,17].

In 2012, Duffis et al. published guidelines suggesting that surgical resection should be performed within one to eight days following embolization [16]. However, more recent recommendations in the literature advocate that preoperative embolization should precede the surgical procedure for 24-48 hours to reduce local edema and inflammation associated with embolization procedures [13,14].

Although several studies have demonstrated benefits from preoperative embolization of the tumor [9,14,15] in terms of minimizing intraoperative complications, two large meta-analyses in 2016 and 2019 [12,18] showed no reduction in blood loss, cranial nerve injury, or vascular injury when surgical resection was performed without preoperative embolization.

Thrombosis of the carotid arterial system, cerebral embolization, stroke, transient ischemic attack, and cranial nerve injuries are major potential risks associated with preoperative embolization [1,13]. However, excellent knowledge of the vascular and surgical anatomy of this region minimizes the incidence of these complications [15]. In our case, the factors that preoperatively guided us to the decision to perform embolization of the tumor vessels were the classification of the tumor according to Shamblin as type II [6], the rich vascularization of the tumor, and its increased size, demonstrated by DSA and CTA preoperatively, because a more complex resection was anticipated on the operating table. We agree with Duffis et al. and Garg et al. that the preference and experience of the senior surgeon were also factors that affected the decision [16,19].

## Conclusions

We present a case in which the proven benefits of preoperative embolization include decreasing tumor vascularity as well as tumor size, which led to limited blood loss during surgery and decreased operating time. We also managed to decrease patient hospitalization time. Although similar results have been demonstrated with only surgical resection of the tumor, preoperative embolization remains a safe and effective tool, especially for large tumors, as demonstrated in our case. A favorable postoperative outcome was observed without neurovascular injury symptoms due to significant assistance from preoperative embolization in the management of this large tumor.
